# The value of miR-155 as a biomarker for the diagnosis and prognosis of lung cancer: a systematic review with meta-analysis

**DOI:** 10.1186/s12885-019-6297-6

**Published:** 2019-11-14

**Authors:** Chuchu Shao, Fengming Yang, Zhiqiang Qin, Xinming Jing, Yongqian Shu, Hua Shen

**Affiliations:** 1 Department of Oncology, The First Affiliated Hospital of Nanjing Medical University, 300 Guangzhou Road, Nanjing, 210029 People’s Republic of China; 2 Department of Oncology, The First Affiliated Hospital of Nanjing Medical University, Nanjing, China; 3 Department of Urology, Nanjing First Hospital, Nanjing Medical University, Nanjing, China

**Keywords:** Lung cancer, miR-155, Diagnosis, Prognosis, Biomarker

## Abstract

**Background:**

Recently, a growing number of studies have reported the coorelation between miR-155 and the diagnosis and prognosis of lung cancer, but results of these researches were still controversial due to insufficient sample size. Thus, we carried out the systematic review and meta-analysis to figure out whether miR-155 could be a screening tool in the detection and prognosis of lung cancer.

**Methods:**

A meta-analysis of 13 articles with 19 studies was performed by retrieving the PubMed, Embase and Web of Science. We screened all correlated literaters until December 1st, 2018. For the diagnosis analysis of miR-155 in lung cancer, sensitivity (SEN), specificity (SPE), positive likelihood ratio (PLR), negative likelihood ratio (NLR), diagnostic odds ratio (DOR) and area under the ROC curve (AUC) were pooled to evaluate the accuracy of miRNA-155 in the diagnosis of lung cancer. For the prognosis analysis of miR-155 in lung cancer, the pooled HRs and 95% CIs of miR-155 for overall survival/disease free survival/progression-free survival (OS/DFS/PFS) were calculated. In addition, Subgroup and meta-regression analyses were performed to distinguish the potential sources of heterogeneity between studies.

**Results:**

For the diagnostic analysis of miR-155 in lung cancer, the pooled SEN and SPE were 0.82 (95% CI: 0.72–0.88) and 0.78 (95% CI: 0.71–0.84), respectively. Besides, the pooled PLR was 3.75 (95% CI: 2.76–5.10), NLR was 0.23 (95% CI: 0.15–0.37), DOR was 15.99 (95% CI: 8.11–31.52) and AUC was 0.87 (95% CI: 0.84–0.90), indicating a significant value of miR-155 in the lung cancer detection. For the prognostic analysis of miR-155 in lung cancer, up-regulated miRNA-155 expression was not significantly associated with a poor OS (pooled HR = 1.26, 95% CI: 0.66–2.40) or DFS/PFS (pooled HR = 1.28, 95% CI: 0.82–1.97).

**Conclusions:**

The present meta-analysis demonstrated that miR-155 could be a potential biomarker for the detection of lung cancer but not an effective biomarker for predicting the outcomes of lung cancer. Furthermore, more well-designed researches with larger cohorts were warranted to confirm the value of miR-155 for the diagnosis and prognosis of lung cancer.

## Background

Lung cancer, as the dominant reason of cancer-associated deaths, remains a serious global public health issue to human beings [1]. Due to lack of effective early screening tools and therapeutic techniques, the clinical outcome of lung cancer patients remains very poor [2]. Thus, a growing number of researchers are commited to finding useful non-invasive biomarkers for cancer detection or predict outcomes, specially in the early stages [3, 4]. However, not all biomarkers have appropirate sensitivity and specificity at the same time like AFP (alpha-fetoprotein), which has been widely applied in hepatocellular carcinoma detection clinically and monitoring development and prognosis of the disease at any time. Consequently, it is imperative to identify a comprehensive biomarker which coluld be used to screen in the early stage of lung cancer or predict clinical outcomes in advance to provide guidance for cancer therapy.

Numerous studies have indicated that microRNAs (miRNAs) are emerging potential biomarkers for cancer detection, predicting clinical outcomes and monitoring disease conditions. MiRNAs refer to short, high conserved, noncoding RNAs that regulate the downstream gene expression in a post-transcriptional manner [5]. Increasing evidences revealed that miRNAs participate in diverse biological processess including cellular multiplication, apoptosis, differentiation, invasion, metastasis, etc. [6]. Moreover, miRNAs are easy to isolate from human body fluids (serum, plasma, etc) combined with excellent stability and non-invasive advantages [7]. Hence, miRNAs might be promising biomarkers in the cancer for early diagnosis, prognosis or clinical treatment responses prediction.

Notably, miR-155 was widely studied as an oncogene involved in multiple cancers [8–12]. Recently, several studies showed that aberrant expression of miR-155 was tied to the diagnosis and prognosis of lung cancer. However, due to different sample sizes, ethnicities and detection methods, these articles showed conflicting results [13]. Hence, this comprehensive meta-analysis was carried out based on previous studies to elaborate the value of miR-155 for lung cancer diagnosis and prognosis.

## Materials and methods

### Search strategy

The systematic literature search was carried out based on PubMed, Embase and other similar databases for eligible original literatures until December 1st, 2018. The relevant keywords “miR-155”, “microRNA-155”, “miRNA-155” and “lung cancer”, “NSCLC”, “lung”, and “prognosis” or “diagnosis” or “detection” or “variants” were used. The MeSH terminology and relevant keywords were randomly combined in order to ensure acquiring the most comprehensive data. In addition, we also sifted through the reference lists of original articles and manually searched from relevant reviews for additional literatures.

### Inclusion and exclusion criteria

In order to screen out eligible studies, specific criteria were used: (1) Research focus on pathological diagnosed lung cancer patients; (2) Detection of miR-155 expression in plasma, serum or other human body fluids; (3) Sufficient data of assessing the coorelation between miR-155 over-expression and poor overall survival (OS), disease free survival (DFS) and progression-free survival (PFS) in lung cancer patients; (4) Available data of true positive (TP), false positive (FP), false negative (FN), true negative (TN) or clear sample size combined with sensitivity (SEN) and specificity (SPE) to calculate the area under the ROC curve (AUC) for diagnostic analysis. In addition, the criteria for patient exclusion were as follows: (1) Studies with no case-control; (2) Non-English or Chinese studies; (3) No data available for lung cancer diagnosis and prognosis; (4) Duplicates or the same samples used in previous publications.

### Data extraction

Two researchers extracted data from all the included studies (SCC and YFM), the uncertain results were assessed by another investigator (QZQ). The extracted data include following information: first author’s name, country, year of publication, ethnicity of the population studied; number of patients and controls; assay type; diagnostic results of SEN, SPE, TP, FP, FN, and TN; or prognostic outcomes including HRs of elevated miR-155 expression for OS/DFS/PFS. Moreover, if not directly available from each article, data was extracted from the Kaplan-Meier curve using the previously described method to infer HR with 95% CI.

### Quality assessment

Two researchers (SCC and YFM) in our institution assessed whether each included literature met the quality standards separately. Then, another researcher (QZQ) reevaluated and make a unified conclusion if there is a discrepancy between first two researchers. For diagnostic meta-analysis, the quality assessment was conducted following the guidelines of the the Quality assessment of diagnostic accuracy studies 2 (QUADAS-2) [14]. This tool include 4 domains to evaluate the risk and applicability of bias, which are refined into 14 specific questions. Each item has a rating of “Yes”, “No” or “Unclear”, corresponding to the scores of − 1, 1 and 0, respectively (Fig. 2). For prognostic meta-analysis, the quality of involved studies were evaluated with the Newcastle-Ottawa Scale (NOS), which is the tool most commonly used to assess the quality of non-randomized research [15, 16]. By scoring one by one, the total quality score ranges from 0 to 9. Studies with a final score > 6 were considered high-quality.

### Statistical analysis

For diagnostic accuracy studies, the SEN, SPE, PLR, NLR and corresponding 95% CI from included studies were pooled to initially assess the diagnostic value of circulating miR-155 in lung cancer. The summary receiver operating characteristic (SROC) curve was then drawn based on the original data, and the area under the SROC curve (AUC) was calculated to comprehensively determine the diagnostic accuracy of miR-155, taking into account the trade-off between SEN and SPE. To assess the heterogeneity across studies, the X^2^-based Q-statistic and I^2^ statistic were utilized. The I^2^ square value typically fluctuates within a range of 0 (unobserved heterogeneity) to 100% (maximum heterogeneity). *P* value < 0.05 or I^2^ > 50% was recognized statistically significant [17]. If the studies were proved to be homogenous, a fixed-effect model would be utilized for further analysis. If not, the random-effect model would be utilized [18]. Subsequently, subgroup and meta-regression analyses were carried out to find the potential sources of heterogeneity. Finally, the publication bias of all the included diagnostic accuracy studies was assessed by Deeks’ funnel plots (significant at *P* < 0.05) [19].

For prognostic meta-analyses, a combination of the pooled HR and 95% CI was calculated to elucidate the link between high expression of miR-155 and cooresponding OS/DFS/PFS of lung cancer patients. Cochran’s Q test and I^2^ statistics were applied to evaluate the heterogeneity of the pooled results [20]. In addition, we used Begg’s and Egger’s tests to assess publication bias. All above statistical analysis was carried out with the statistical software STATA (version 12.0) [21].

## Results

### Litereture search results

**Fig. 1:**
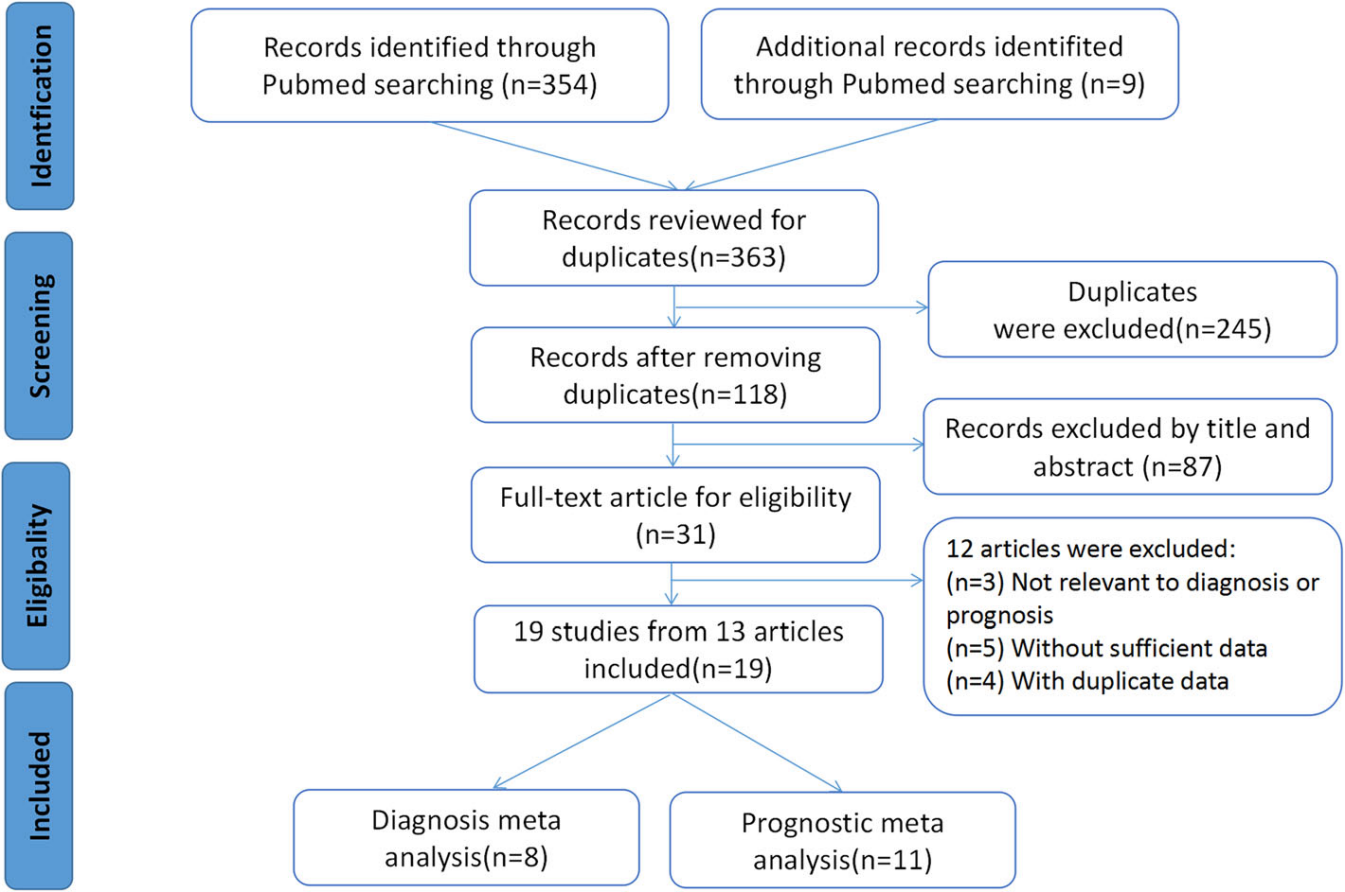
Flow chart of selection process

Based on a systematic search on the above databases, 363 records related to miR-155 in lung cancer were initially identified. Then, 245 duplicates were deleted following the inclusion and exclusion criteria described previously. Eighty-seven articles were subsequently removed after a quick skim through the titles and abstracts. As a result, the remaining 31 articles were all downloaded to obtain valid information individually. After reading the full texts carefully, 12 studies were elimated due to lack of available diagnostic or prognostic related data. Ultimately, this meta-analysis included 13 articles covering 19 cohort studies [22–34]. Among them, 6 articles with 8 studies focused on the miR-155 expression for lung cancer diagnostic accuracy, whereas 7 articles including 11 studies related to the correlation of miR-155 and lung cancer prognosis. **(**Fig. 1**).**

### Studies characteristics and quality assessment

**Fig. 2:**
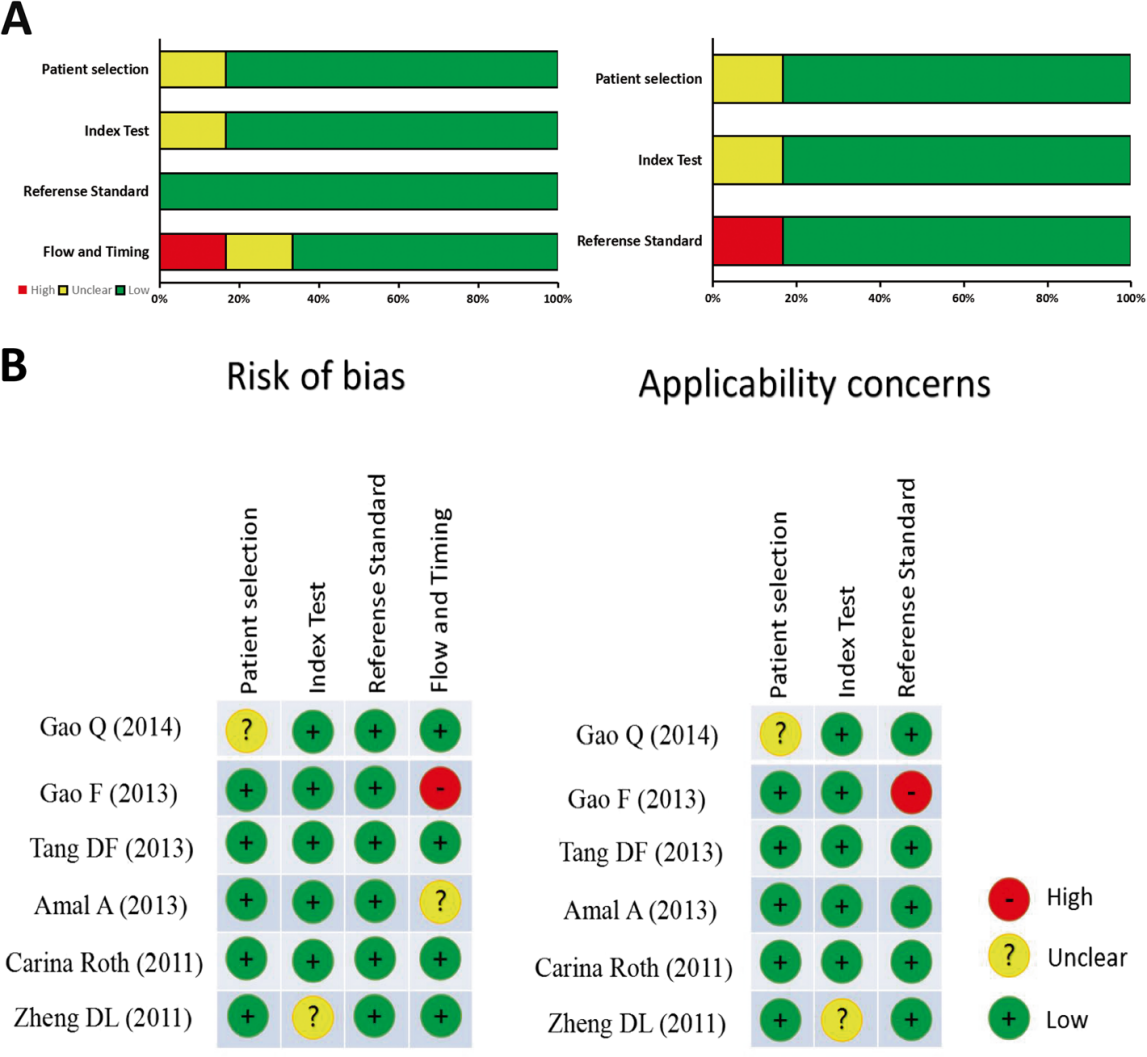
QUADAS-2 quality assessment. Investigators’ assessment regarding each domain for included studies: (**a**) The graph and (**b**) summary

In 8 eligible studies for diagnostic analysis, 457 cases and 342 controls were identified as presented on Table 1. Among these 8 studies, three ethnic groups were analyzed, in which six from Asians, one from Africans, and the remaining from Caucasians. All included studies detected miR-155 expression through qRT-PCR using SYBR or Tagman reagent. The results of QUADAS-2 quality assessment were shown in Fig. 2a and 2b. Most studies were consistent with the criteria in QUADAS-2, indicating that the enrolled studies are suitable for quantitative synthesis.

**Table 1:** Characteristics and methodology assessment of 8 studies included in the diagnosis meta-analysis

First author	Year	Country	Ethnicity	Case/Control	Assay type	SEN (%)	SPE(%)	TP	FP	FN	TN
Feng Gao [22]	2013	China	Asian	36/32	SYBR	72.20	68.70	26	8	10	22
Dongfang Tang (1) [23]	2013	China	Asian	62/60	TaqMan	59.70	75.00	37	15	25	45
Dongfang Tang (2) [23]	2013	China	Asian	34/32	TaqMan	67.60	65.60	23	11	11	21
Qing Geng (1) [24]	2014	China	Asian	25/25	SYBR	87.00	87.00	22	3	3	22
Qing Geng (2) [24]	2014	China	Asian	126/60	SYBR	86.00	84.00	108	10	18	50
Amal A [25].	2013	Egypt	African	65/37	SYBR	95.40	62.20	62	14	3	23
Carina Roth [26]	2011	Germany	Caucasian	35/28	TaqMan	87.70	88.90	31	3	4	25
Dali Zheng [27]	2011	China	Asian	74/68	SYBR	80.36	83.93	59	11	15	57

In the 7 included articles for prognosis, a total of 1382 participants were identified for assessing OS/DFS/PFS, respectively. The characteristics of these enrolled literatures were presented on Table 2. The included population were classified into Asians and Caucasians from five different countries, including China, France, America, Japan and Norway. In addtion, the detailed quality assessment for each study scored following the guidlines of NOS is shown in Table 3.

**Table 2:** The main features of 11 included studies in prognostic meta-analysis

First author	Year	Country	Ethnicity	Case	Outcome	HR (95%CIs)	P value	
Mitch Raponi [28]	2009	America	Caucasian	54	OS	2.30 (1.00–5.60)	0.060	
Motonobu Saito (1) [29]	2011	Japan	Caucasian	89	PFS	2.37 (1.27–4.42)	0.006	
Motonobu Saito (2) [29]	2011	Japan	Caucasian	37	PFS	1.60 (0.73–3.52)	0.245	
Motonobu Saito (3) [29]	2011	Japan	Asian	191	PFS	1.33 (0.77–2.29)	0.309	
Yi Gao [30]	2014	China	Asian	162	OS	2.31 (1.48–3.61)	< 0.001	
Johannes Voortman [31]	2010	France	Caucasian	637	OS	0.91 (0.72–1.13)	0.390	
Tom Donnem (1) [32]	2011	Norway	Caucasian	191 (SCC)	PFS	0.45 (0.21–0.96)	0.039	
Tom Donnem (2) [32]	2011	Norway	Caucasian	95 (AC)	PFS	1.87 (1.01–3.48)	0.047	
Ce´ line Sanfiorenzo [33]	2013	France	Caucasian	52	DFS	0.94 (0.15–5.74)	0.008	
Xinying Xue (1) [34]	2016	China	Asian	80	OS	0.52 (0.24–1.14)	0.045	
Xinying Xue (2) [34]	2016	China	Asian	80	DFS	0.83 (0.30–2.31)	0.054	

OS: overall survival; DFS: disease free survival; PFS: progression-free survival;SCC:squamous cell carcinoma;AC:Adenocarcinoma

**Table 3:** Newcastle–Ottawa quality assessments scale

First author	Year	Quality indicators from Newcastle–Ottawa Scale								Scores
		1	2	3	4	5	6	7	8	
Raponi [28]	2009	★	★	–	–	★★	★	★	★	7
Saito [29]	2011	★	★	–	★	★★	★	★	★	8
Yi G [30]	2014	★	★	–	–	★★	★	★	★	7
Voortman [31]	2010	–	–	–	★	★★	★	★	★	6
Donnem [32]	2011	★	–	–	–	★★	★	★	★	6
Sanfiorenzo [33]	2013	★	★	–	★	★★	★	★	★	8
Xue [34]	2016	★	★	–	★	★★	★	★	–	7

1. Representativeness of the exposed cohort; 2. Selection of the non-exposed cohort; 3. Ascertainment of exposure; 4. Outcome of interest not present at start of study; 5. Control for important factor or additional factor; 6. Assessment of outcome; 7. Follow-up long enough for outcomes to occur; 8. Adequacy of follow up of cohorts

### Diagnosis meta-analysis

#### Pooled diagnostic value of miR-155 in lung cancer

**Fig. 3:**
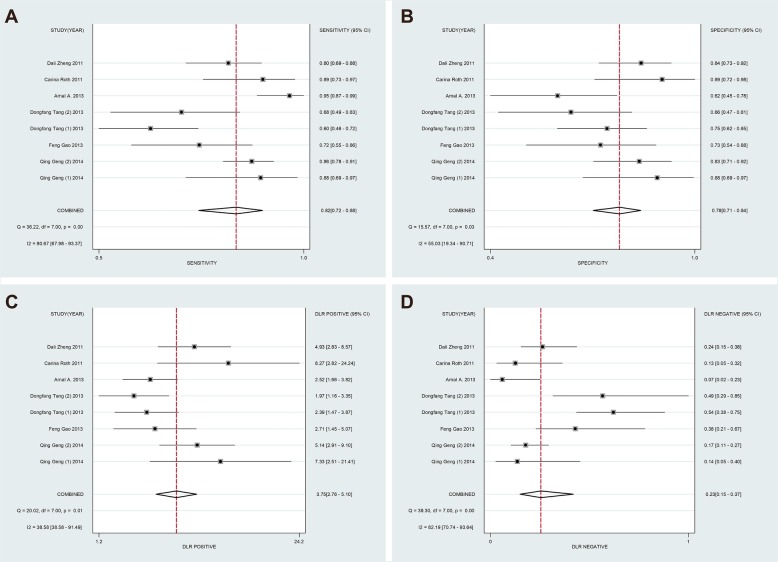
Forest plots of sensitivity (**a**), specificity (**b**), positive likelihood ratios (**c**) and negative likelihood ratios (**d**) for miR-155 in the diagnosis of lung cancer

The forest plots results were presented in Fig. 3a and 3b as follows: the pooled SEN and SPE were 82% (95% CI: 78–88%) and 78% (95% CI: 71–84%). The PLR and NLR were 3.75 (95% CI: 2.76–5.10) and 0.23 (95% CI: 0.15–0.37) respectively (Fig. 3c and 3d). Meanwhile, the pooled DOR was 15.99 (95% CI: 8.11–31.52) (Fig. 5a) and the area under SROC (AUC) was 0.85 (95% CI: 0.82–0.88) (Fig. 6a). All above data demonstrated the relatively high diagnostic value of miR-155 in lung cancer.

#### Subgroup analysis

**Fig. 4:**
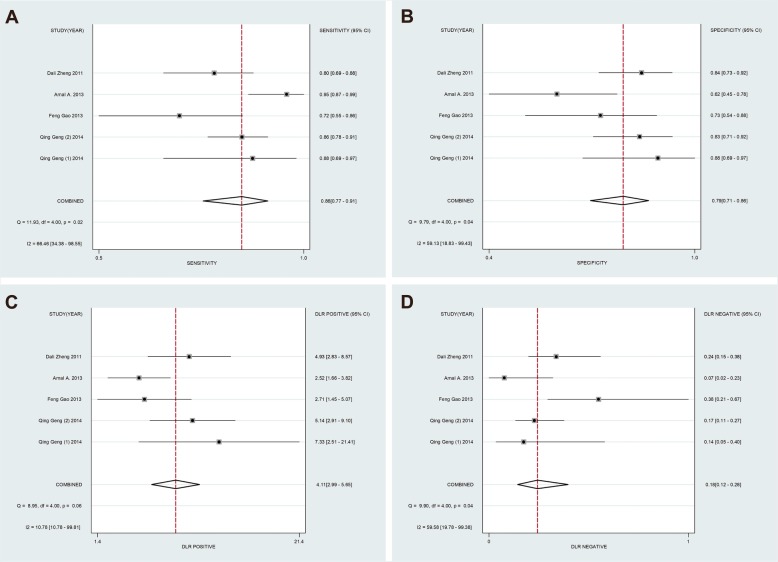
Subgroup analysis based on Assay type of sensitivity (**a**), specificity (**b**), positive likelihood ratios (**c**) and negative likelihood ratios (**d**) for miR-155 by SYBR in the diagnosis of lung cancer

**Fig. 5:**
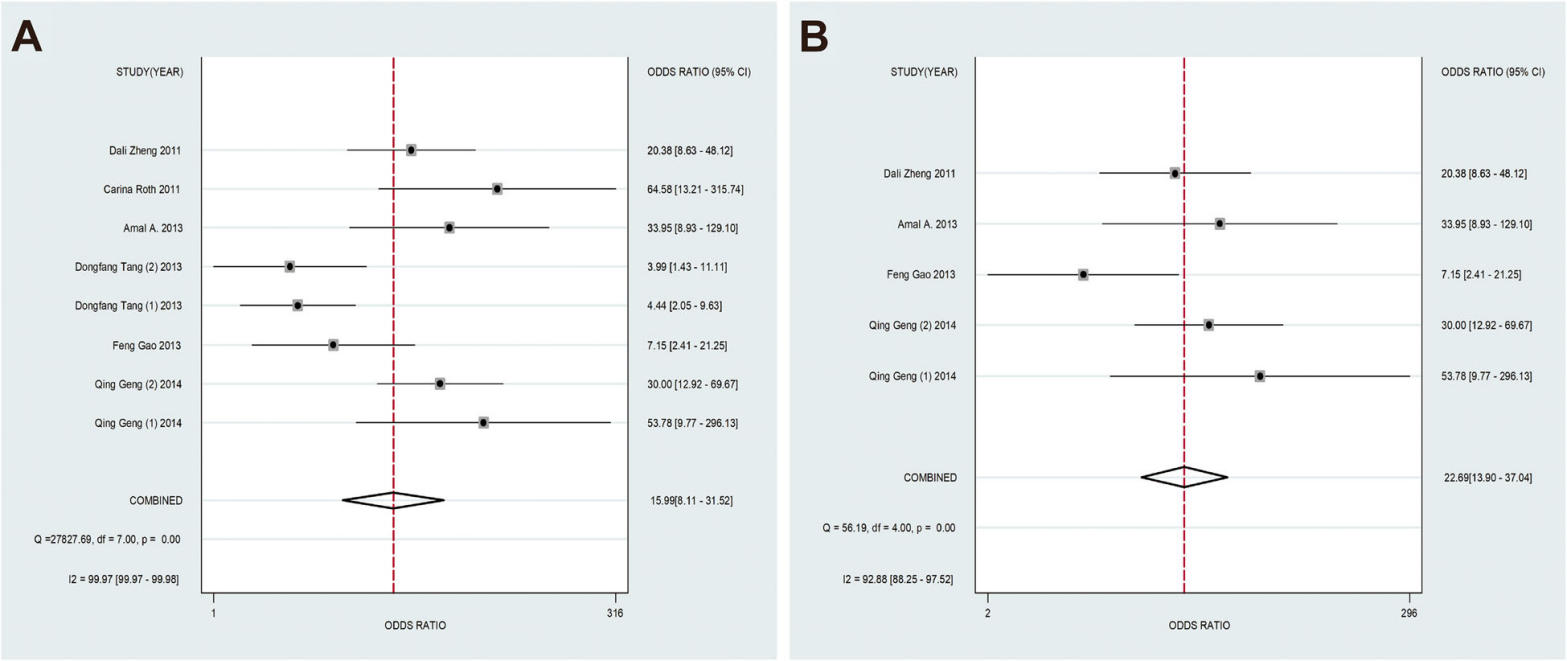
Forest plots of the diagnostic odds ratio (DOR) for miR-155 in the diagnosis of lung cancer. (**a**). All studies; (**b**). The studies based on SYBR

**Fig. 6:**
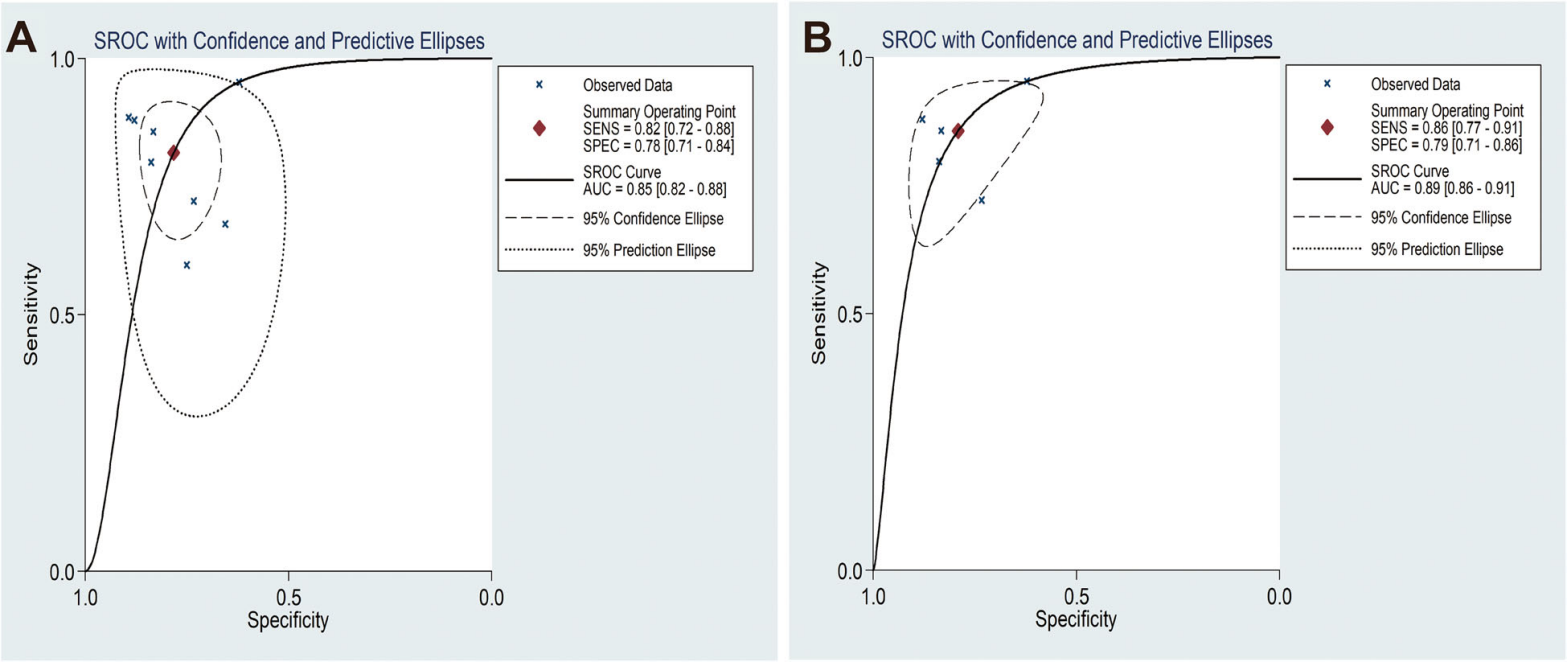
Summary receiver operating characteristic curves (sROC) from the hierarchical summary receiver operating characteristic model generated from the 8 studies that found that miR-155 was a diagnostic marker for lung cancer. (**a**). All studies; (**b**). The studies based on SYBR

To distinguish the potential origins of heterogeneity between studies, a subgroup analysis was perfomed based on Assay type. The pooled results of this subgroup analysis were shown in Fig. 4. It can be observed that studies based on SYBR qPCR method showed similar results: the SEN was 86% (95% CI: 77–91%), SPE was 79% (95% CI: 71–86%), PLR was 4.11 (95% CI: 2.99–5.65) and NLR was 0.18 (95% CI: 0.12–0.28), respectively. The summary DOR was 22.69 (95% CI: 13.90–37.04) (Fig. 5b) and AUC was 0.89 (95% CI: 0.86–0.91) (Fig. 6b).

### Prognosis meta-analysis

**Fig. 7:**
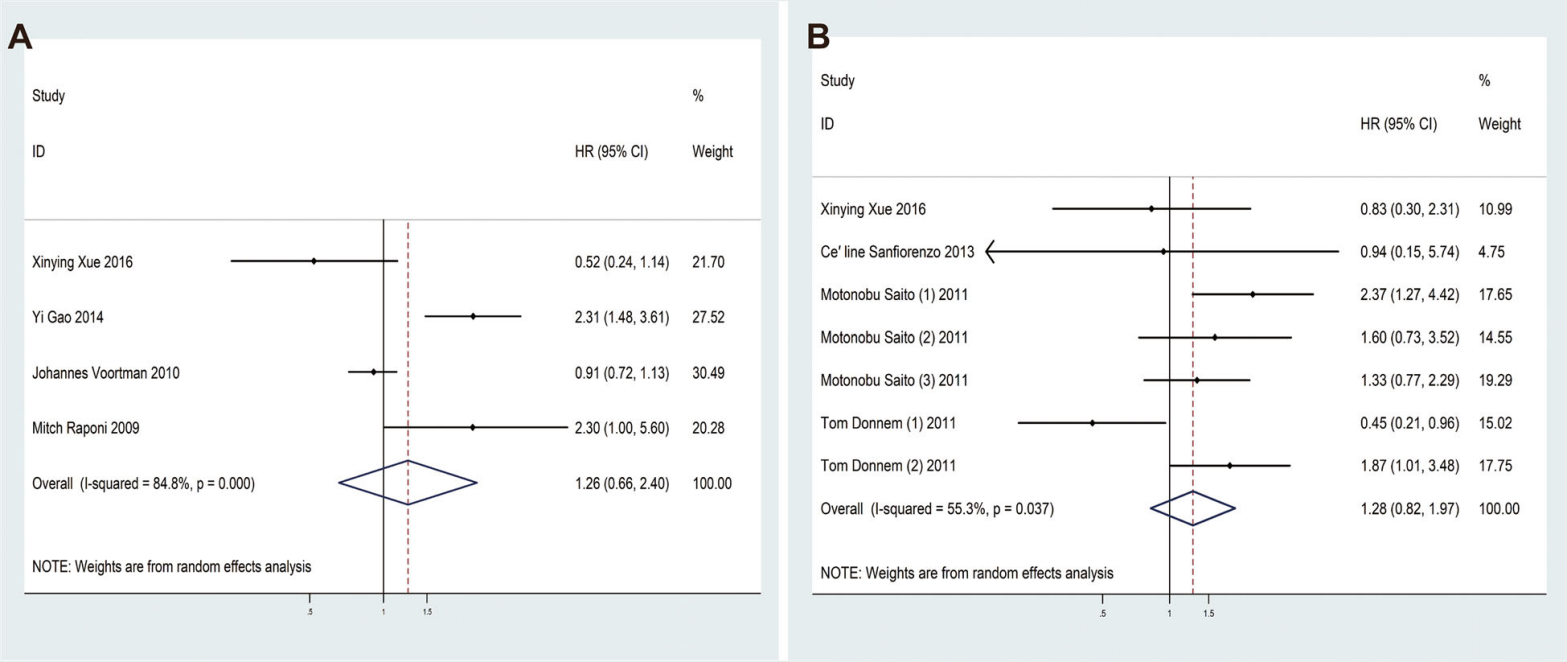
Forest plots of the studies that evaluated the hazard ratios of high miR-155 expression. (**a**). The studies based on OS; (**b**). The studies based on DFS/PFS

The main outcome of the prognostic meta-analysis was to evaluate the correlation between miR-155 expression and OS/DFS/PFS of lung cancer patients. In the 4 studies evaluating OS, the pooled HR and its 95% CIs were calculated using a random-effect model with a result of 1.26 (95% CI: 0.66–2.40) (Fig. 7a). Meanwhile, for 7 studies evaluating DFS/PFS, the combined HR with 95% CIs was 1.28 (95% CI: 0.82–1.97) (Fig. 7b). To sum up, the results given above proved that there was not significant correction between over-expression of miRNA-155 and poor OS or DFS/PFS.

### Publication bias and meta-regression analyses

The potential publication bias across the enrolled diagnostic studies was accessed by the Deeks’ funnel plot test whereas the prognostic studies evaluated using Begg’s funnel plot and Egger’s test. The Deeks’ funnel plot was symmetry and reached a *P* value of 0.951 above 0.05, indicating there is no obvious publication bias in these included studies. The *P* values of Begg’s tests for OS and DFS/PFS were 0.497 and 0.453. The results of Egger’s test (OS: *P* = 0.785, DFS/PFS: *P* = 0.264, respectively) also proved no existence of publication bias. These results indicated that the data were reliable in the current meta-analysis.

## Discussion

As a malignant tumor with extremely high mortality, lung cancer has gaining great attention and extensive researches during recent decades. With the development of surgical techniques, concurrent radiotherapy and chemotherapy, and imaging examination technology have greatly improved the prognosis of lung cancer patients. Nevertheless, the most effective way to improve the survival of lung cancer patients lies in early diagnosis and targeted treatment. Therefore, a large amount of researchers are committed to finding suitable non-invasive biomarkers to predict the diagnosis or prognosis of lung cancer, and provide directions for clinical treatment of lung cancer .

As vital regulators of various biological processes in cancer, miRNAs were regarded as perfect non-invasive biomarkers for human cancers [35, 36]. MiR-155 was widely studied to participate in the occurrence and progression of diverse cancers, including lung cancer [37]. Several researches suggested that up-regulated miR-155 is positively correlated with the pathogenesis of lung cancer, indicating that miR-155 acts as an oncogene in lung cancer [37, 38]. Zang et al. revealed that miR-155 was involved in the drug resistance of lung cancer. In their study, miR-155 was shown to modulate celluar poptosis and DNA damage via Apaf-1 regulated pathways to decrease the sensitivity of lung cancer cells to cisplatin [38]. Moreover, another research conducted by Katrien et al. found that miR-155 increases resistance to chemotherapy in lung cancer cells by forming a feedback loop with TP53 [39]. In particular, they also found that over-expression of miR-155 is significantly linked to poor OS of lung cancer patients. These results indicated that miR-155 has the potential to be an ideal biomarker for lung cancer. In addition to the above studies focused on the molecular mechanism of miR-155 regulation in lung cancer cells, accumulating cohort studies have reported the coorelationship between miR-155 levels in different individuals with lung cancer diagnosis or prognosis to determine whether miR-155 acts as an ideal biomarker [40, 41]. However, these results have not been corroborated and even contradictory. Thus, this meta-analysis appears to be necessary to figure out the diagnostic and prognostic value of miR-155 for lung cancer.

In the diagnositic meta-analysis, the total DOR with 95% CI of miR-155 was 15.99 (95% CI: 8.11–31.52). In addition, AUC and corresponding 95% CI were 0.85 (95% CI: 0.82–0.88), indicating that miR-155 could act as a moderate marker in the lung cancer diagnosis compared to healthy individuals. Subgroup analysis of Assay type revealed that studies based on SYBR had a higher DOR of 22.69 (95% CI: 13.90–37.04) and the higher AUC of 0.89 (95% CI: 0.86–0.91), which might be the possible sources of heterogeneity. Nowadays, several tumor biomarkers have been applied for detecting early lung cancer clinically, such as CA-125, CEA, CYFRA21-1, NSE and so on. However, limited sensitivity and specificity of these existing biomakers restricted their diagnostic accuracy. Based on the 6 included articles, miR-155 can be stably detected in the plasma of lung cancer patients with marked differences when compared with control samples, suggesting it can serve as a serum-based biomarker for lung cancer detection individually. As Currently, miR-155 hasn’t been applied as a clinical diagnostic tool in patients that had not previously been diagnosed with lung cancer. And clinical detection of lung cancer usually involves not only a single miRNA, but a combination of miRNAs.What’s more, miR-155 could be combined with traditional biomarkers for the diagnosis of lung cancer, so as to improve the diagnosis accuracy in the future.

By the way, as polymorphisms in genes encoding miRNAs may alter the expression of the corresponding miRNA and thus confer susceptibility to multiple diseases such as cancers, it might be meaningful to investigate the association between polymorphisms in genes encoding miR-155 and lung cancer susceptibility. For example, previous study published by Xie et al. identified that rs767649 (A > T) in regulatory regions of miR-155 was associated with the increased risk and poor prognosis of lung cancer [42]. What’s more, they found four target genes of miR-155 including *HBP1, TJP1, SMAD5* and *PRKAR1A* involved in the oxidative stress process of lung cancer. Given that miR-155 is a typical oncogene in lung cancer, more well-designed studies in the future could confirm its diagnostic value, and more importantly, further researches colud focus on gene polymorphisms encoding miR-155, which can manually regulate miRNA levels, leading to changes in cancer-associated downstream protein signaling pathways.

On the other hand, the prognostic meta-analysis suggested that up-regulated miR-155 might not be associated with poor clinical outcomes of lung cancer patients, which was 1.26-fold higher risk for poor OS and 1.28-fold higher risk for poor DFS/PFS. These results might caused by different genetic backgrounds, environmental exposures and detection methods. Recently, accumulating studies worldwide have shown that expression levels of miRNAs in different individuals have significant predictive value in cancers. Currently, the detection of miRNAs in tissue samples has been applied to current tumor prognosis studies, but the detection of serum/plasma samples and other human body fluids appears to be more portable, non-invasive, and can effectively assess survival prognosis at any time before or after treatment. It can even play a role in the patient’s life-long disease surveillance and is of great help to clinical thearapy. This meta-analysis found that miR-155 has no obvious prognostic effect on lung cancer, which is inconsistent with results of some previous prognostic studies, while the result is consistent with the prognostic value of miR-155 of NSCLC reported in a meta-analysis published by Lamichhane SR et al. in 2018 [43]. However, the sample size included in our meta-analysis is larger than previous mata-analysis, more researches with sufficient data will be needed to verify this result.

Ultimately, several limitations still existed in this meta-analysis as follows: (1) Racial factors were not comprehensive enough, and the population is too monotonous. For example, the diagnostic meta-analysis is mainly for Asians and Africans while the prognostic meta-analysis only focused on Caucasians and Asians. Therefore, more researchers should pay attention to the impact of racial factors in the subsequent studies. (2) Unpublished studies may contain negative results, but we are not available include them, which potentially lead to lack of credibility in the data. (3) We only included articles published in English and Chinese, but did not cover articles in other languages. (4) The sample size was still relatively small, including only 19 studies, which may undermine the reliability of our findings. Therefore, more well-designed studies based on larger samples and sufficient data are required to verify the diagnostic and prognostic value of circulating miR-155 in lung cancer. (5) Adjusted estimates could not be performed in our meta analysis without enough data for the adjustment by other covariates such as TNM stage, histological type, mean of age, gender and so on.. Therefore, further high-quality researches in the risk of lung cancer might be performed to draw more accuracy results in subsequent years.

## Conclusion

To summarize, our meta-analysis demonstrated for the first time that circulating miR-155 is promising to be a novel biomarker for diagnosis of lung cancer. However, miR-155 is not an effective biomarker for predicting the prognosis of lung cancer. Together, these findings provide important evidence for further development of future non-invasive methods for diagnosing lung cancer. Further large-scale relevant studies with better designs and more comprehensive data support will help to clarify the diagnostic and prognostic value of miR-155 in lung cancer.

## Data Availability

The data that support the findings of this study are available from the corresponding author upon reasonable request.

## Abbreviations

AUC: Area under the ROC curve
DFS: Disease free survival
DOR: Diagnostic odds ratio
FN: False negative
FP: False positive
HR: Hazard ratio
miRNAs: MicroRNAs
NLR: Negative likelihood ratio
NOS: Newcastle-Ottawa Scale
OS: Overall survival
PFS: Progression-free survival
PLR: Positive likelihood ratio
QUADAS: Quality assessment of diagnostic accuracy studies
TN: True negative
TP: True positive
